# Supplementary material for: The Influence of the Preparation Method on the Physico-Chemical Properties and Catalytic Activities of Ce-Modified LDH Structures Used as Catalysts in Condensation Reactions
Source: Molecules. 2021 Oct 14;26(20):6191. doi: 10.3390/molecules26206191 (PMC8537870; doi:10.3390/molecules26206191)
Supplement: Supplementary file 1 [file molecules-26-06191-s001.zip › molecules-1422622-supplementary.pdf]

Electronic Supplementary Information

The influence of the preparation method on the physico-chemical properties and catalytic activities of Ce-modified LDH structures used as catalysts in condensation reaction

Alexandra-Elisabeta Stamate [1,2], Rodica Zăvoianu [1,2*], Octavian Dumitru Pavel [1,2], Ruxandra Bîrjega [3], Andreea Matei [3], Marius Dumitru [3], Ioana Brezeștean [4,5], Mariana Osiac [6] and Ioan-Cezar Marcu [1,2*]

1 Department of Organic Chemistry, Biochemistry and Catalysis, Faculty of Chemistry, University of Bucharest, 4-12, Blvd. Regina Elisabeta, 030018 Bucharest, Romania; alexandra-elisabeta.stamate@drd.unibuc.ro (A.-E.S.); octavian.pavel@chimie.unibuc.ro (O.D.P); rodica.zavoanu@chimie.unibuc.ro (R.Z.); ioancezar.marcu@chimie.unibuc.ro (I.-C.M.)

2 Research Center for Catalysts and Catalytic Processes, Faculty of Chemistry, University of Bucharest, Bucharest 030018, Romania

3 National Institute for Lasers, Plasma and Radiation Physics, 409 Atomistilor Street, P.O. Box MG-36, 077125 Magurele, Romania; email: ruxandra.birjega@inflpr.ro (R.B.), marius.dumitru@inflpr.ro (M.D.), andreea.purice@inflpr.ro (A.M.)

4 National Institute for R&D of Isotopic and Molecular Technologies, 67-103 Donat, 400293 Cluj-Napoca, Romania,

5 Babes-Bolyai University, Faculty of Physics, Biomolecular Physics Department, Cluj-Napoca 400084, Romania (PhD student); ioana.brezestean@itim-cj.ro

6 University of Craiova, Department of Physics, INCESA Craiova 200585, Romania; mariana71osiac@gmail.com

* Correspondence: rodica.zavoianu@chimie.unibuc.ro (R.Z.); ioancezar.marcu@chimie.unibuc.ro (I.-C.M); Tel.: +40-746-171-699 (R.Z.); +40-213-051-464 (I.-C.M)

XPS spectra

An Escalab Xi+ system, Thermo Scientific (Waltham, MA, USA) was used for X-ray photoelectron spectroscopy (XPS) survey and high-resolution XPS spectra acquisition. The survey scans were acquired using an Al Kα gun with a spot size of 900 μm, pass energy of 10.0 eV and an energy step size of 1.00 eV (5 scans). 20 scans were accumulated for Ce3d high-resolution XPS spectra, the pass energy was set to 10.0 eV, and the energy step size was 0.10 eV. The spectra can be seen below:

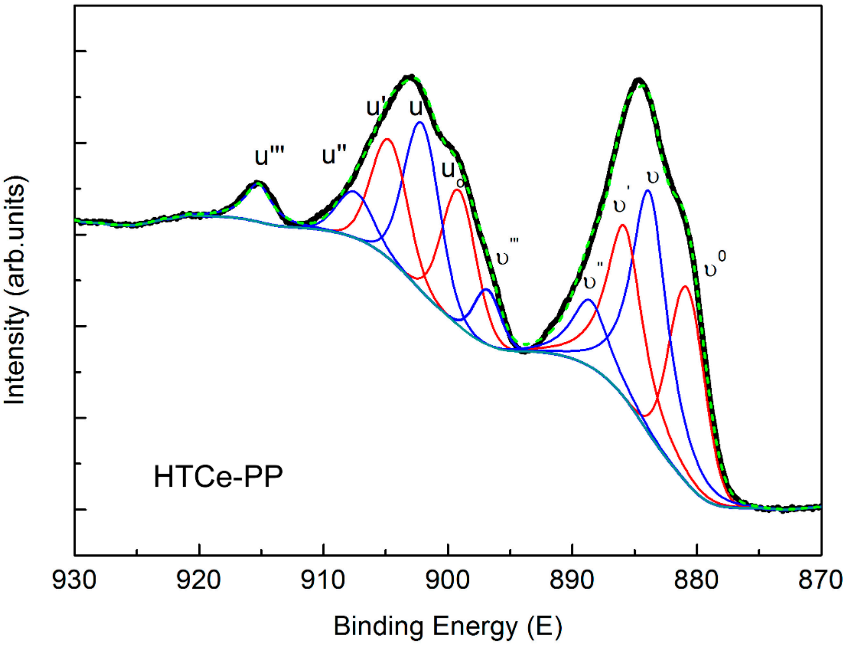

(a)

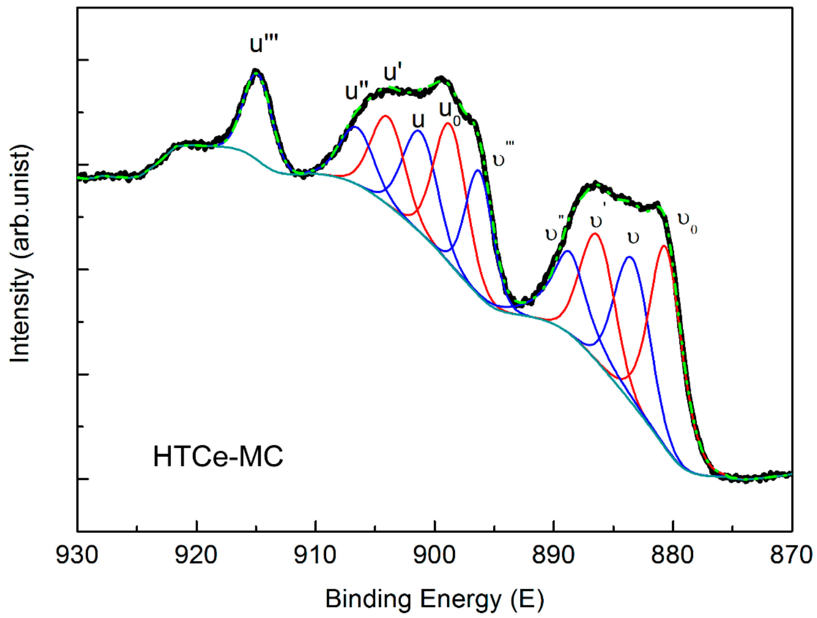
u'''
u''
u'
u
u0
υ'''
υ''
υ'
υ
υ0
HTCe-MC
930
920
910
900
890
880
870
Binding Energy (E)
Intensity (arb.unist)

(b)

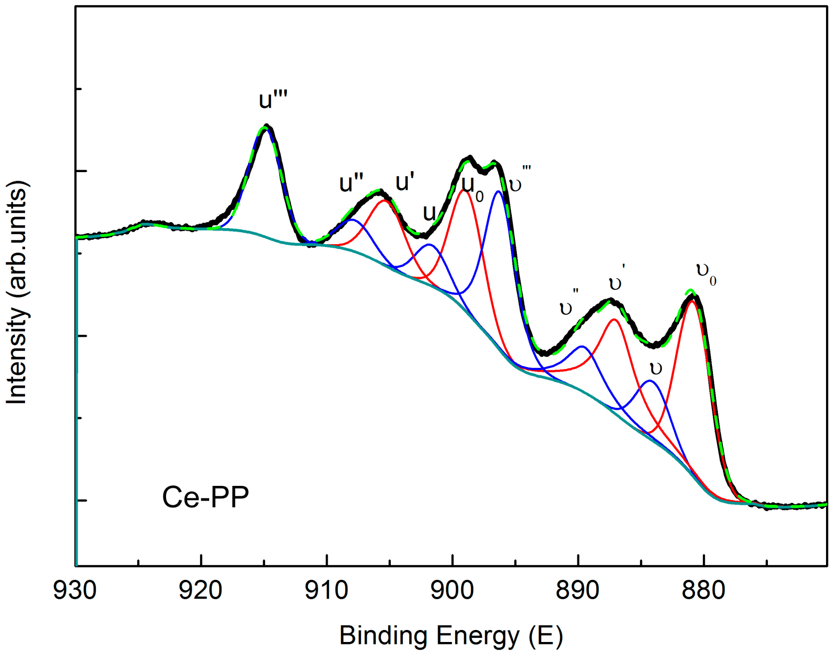
u'''
u''
u'
u
u0
υ'''
υ''
υ'
υ
υ0
Ce-PP
930
920
910
900
890
880
Binding Energy (E)
Intensity (arb.units)

(c)

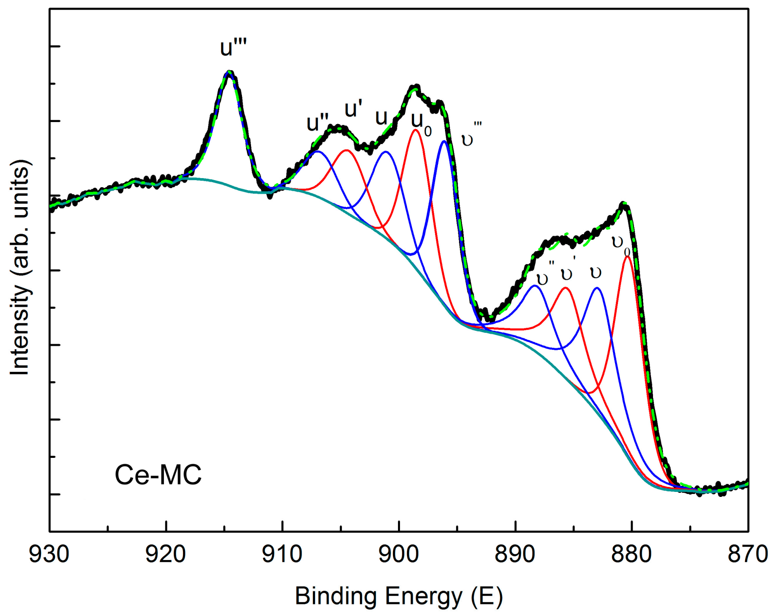

(d)

Figure S1. Ce3d high-resolution XPS spectra for Ce-containing LDH catalysts: a) HTCe-PP, b) HTCe-MC, c) Ce-PP, d) Ce-MC
